# Assessing the recovery process in a mobile rehabilitation team for people with severe mental disorders by using the Recovery Helm

**DOI:** 10.1192/j.eurpsy.2024.477

**Published:** 2024-08-27

**Authors:** S. Strkalj Ivezic, M. Curkovic, D. Štimac Grbić

**Affiliations:** ^1^Research, University psychiatric hospital Vrapce; ^2^Department for mental helat, Croatian institute for public healht; ^3^School of public health A Stampar, School of Medicine University of Zagreb, Zagreb, Croatia

## Abstract

**Introduction:**

People with severe mental disorder (SMD) determine the goals and paths of recovery with professional and nonformal supporters such as family and friends. It is crucial that these people, as well as everyone who participates in the recovery process, are familiar with all the elements that contribute to the recovery of mental health in order to help people with SMD identify goals and assess their achievement. We have therefore created a Recovery Helm to assess the functioning in the various areas necessary for recovery to help us assess the needs and monitor the recovery process of people with mental health problems.

**Objectives:**

The goal is to assess the initial state of mental health and monitor the effects of the mobile rehabilitation team program on the recovery of people with SMI through the use of the Recovery Helm.

**Methods:**

We used the Recovery Helm: http://shorturl.at/gyCDQ as an instrument for the initial assessment of all areas crucial for recovery to determine the goals of recovery and interventions needed to achieve these goals of rehabilitation in 30 patients included in the program of the mobile rehabilitation team applying different psychosocial interventions according to the individual recovery plan made as a mutual agreement between patients and rehabilitation team. The status of recovery is evaluated after 3 and 6 months.

**Results:**

The results indicate significant improvements in most areas of the recovery assessed at the Recovery Helm selected as individually important goal for a person included in the rehabilitation program

**Conclusions:**

The Recovery Helm is an excellent clinical assessment instrument that helps determine recovery goals and rehabilitation interventions that promote recovery and monitor the achieved results.

**Disclosure of Interest:**

None Declared

